# Posterior Percutaneous Pedicle Screws Fixation Versus Open Surgical Instrumented Fusion for Thoraco-Lumbar Spinal Metastases Palliative Management: A Systematic Review and Meta-analysis

**DOI:** 10.3389/fonc.2022.884928

**Published:** 2022-04-04

**Authors:** Andrea Perna, Amarildo Smakaj, Raffaele Vitiello, Calogero Velluto, Luca Proietti, Francesco Ciro Tamburrelli, Giulio Maccauro

**Affiliations:** ^1^ Department of Geriatrics and Orthopaedic Sciences, Università Cattolica del Sacro Cuore, Rome, Italy; ^2^ Department of Aging, Neurological, Orthopaedic and Head-Neck Sciences, Fondazione Policlinico Universitario Agostino Gemelli Istituto di Ricovero e Cura a Carattere Scientifico (IRCCS), Rome, Italy

**Keywords:** spinal metastasis, cancer surgery, minimally invasive spine surgery, MIS, percutaneous pedicle screws

## Abstract

**Background:**

Surgical palliative treatment of spinal metastases (SM) could influence the quality of life (QoL) in cancer patients, since the spine represents the most common site of secondary bony localization. Traditional open posterior instrumented fusion (OPIF) and Percutaneous pedicle screw fixation (PPSF) became the main surgical treatment alternatives for SM, but in Literature there is no evidence that describes the absolute superiority of one treatment over the other.

**Materials and Methods:**

This is a systematic review and meta-analysis of comparative studies on PPSF versus OPIF in patients with SM, conducted according to the Preferred Reporting Items for Systematic Reviews and Meta-Analyses (PRISMA) statement. The outcomes of interest were: complications, blood loss, infections, mortality, pain and also the Quality of Life (QoL).

**Results:**

There were a total of 8 studies with 448 patients included in the meta-analyses. Postoperative complications were more frequent in OPIF (odds ratio of 0.48. 95% CI, 0.27 to 0.83; p= 0.01), PPFS was associated with blood loss (odds ratio -585.70. 95% IC, -848.28 to -323.13.69; p< 0.0001) and a mean hospital stay (odds ratio -3.77. 95% IC, -5.92 to -1.61; p= 0.0006) decrease. The rate of infections was minor in PPFS (odds ratio of 0.31. 95% CI, 0.12 to 0.81; p= 0.02) whereas the occurrence of reinterventions (0.76. 95% CI, 0.25 to 2.27; p= 0.62) and the mortality rate was similar in both groups (odds ratio of 0.79. 95% CI, 0.40 to 1.58; p= 0.51). Finally, we also evaluated pre and post-operative VAS and the meta-analysis suggested that both techniques have a similar effect on pain.

**Discussion and Conclusion:**

The PPSF treatment is related with less complications, a lower rate of infections, a reduction in intraoperative blood loss and a shorter hospital stay compared to the OPIF treatment. However, further randomized clinical trials could confirm the results of this meta-analysis and provide a superior quality of scientific evidence.

## Introduction

The bone represent the third most frequent secondary cancer location, after lung and liver, especially for solid tumour such as lung, prostate and breast (1). Spinal metastases (SM) is the most frequent metastatic bone lesion (MBL) and one of the principal causes of morbidity and worsening of the quality of life (QoL) in cancer patients due to neurologic involvement and intractable pain (2). It is estimated that about 10% of cancer patients have symptomatic SM, and the thoraco-lumbar region seems to be the most involved (3). Furthermore the life expectancy of cancer patients increased, and consequently both the incidence and prevalence of symptomatic SM represents growing condition (4). Often the correct management of SM is challenging for doctors. The SM patient treatment must be individualized for each patient, requiring a multidisciplinary approach among the various medical specialists (5).

Several therapeutic alternatives were described such as chemotherapy or radiotherapy, however surgery seems to be the best choice for spinal instability related pain and neurological impairment (6). The presence of SM often reflects an advanced disease where is not possible for a spinal surgeon to be radical. Therefore palliative surgery, with the aim of improving the patient’s QoL for the remaining life, represents an increasingly occurrence in spinal oncology (7).

Traditional open posterior instrumented fusion (OPIF) with or without decompression was described as effective in the neurological status improvement. However, the high rate of peri and post-operative complications could affect the final outcome and consequently the patients’ QoL (8). Percutaneous pedicle screw fixation (PPSF) advantages (reduced blood loss, less soft tissue trauma, less perioperative pain, shorter hospitalization and earlier return to normal activities) were widely reported in polytrauma patients and degenerative spinal diseases (9). Nevertheless, only in recent years, the use of minimally invasive spinal surgery (MISS) in SM patients has increased. Currently in Literature there is no evidence that indicates the absolute superiority of one treatment over the other (10). Therefore, the aim of the present systematic review and meta-analysis was to evaluate PPSF versus OPIF approaches in treatment of SM patients.

## Materials and Methods

### Study Setting and Search Strategy

**Figure 1:**
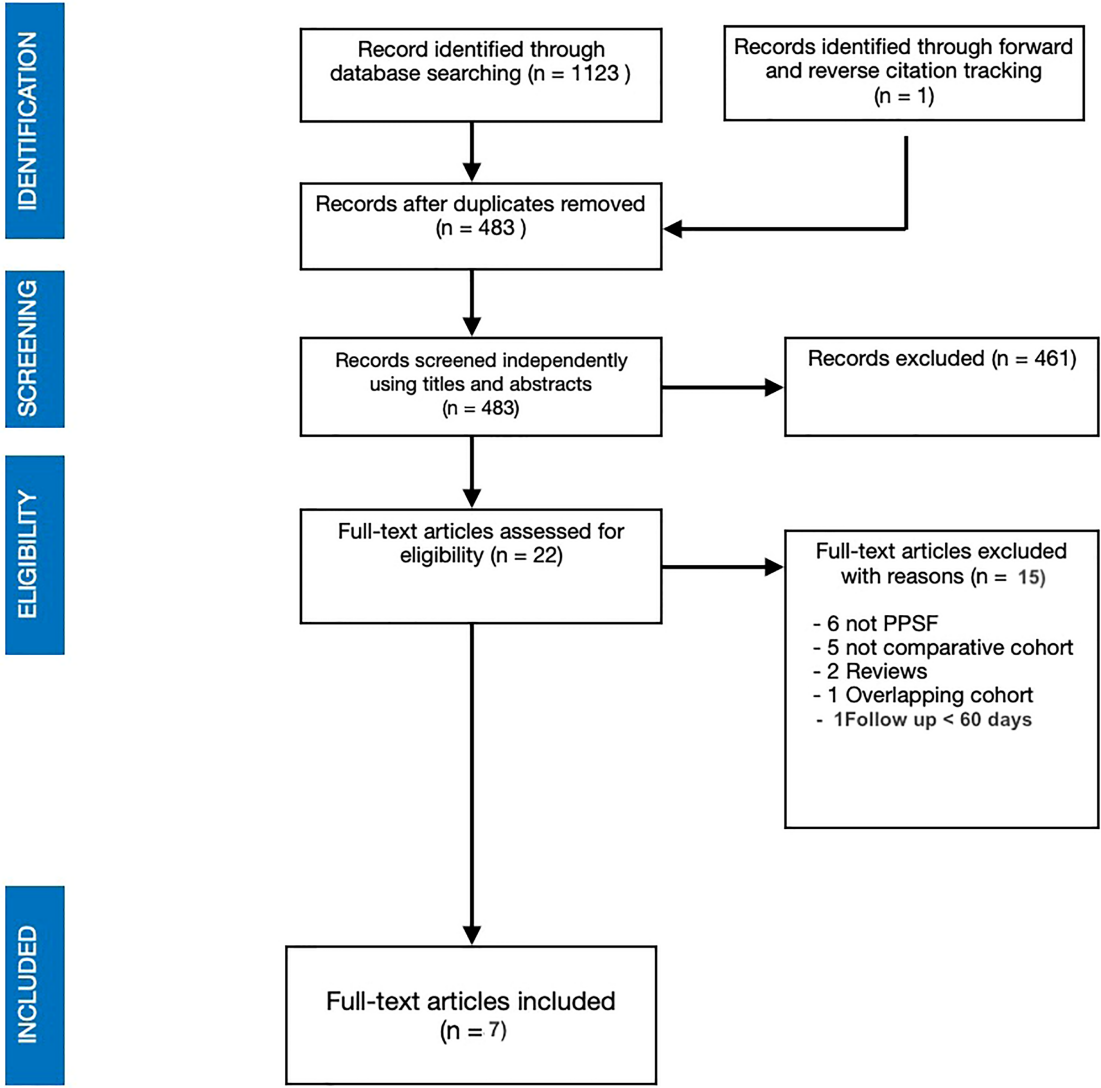
PRISMA search strategy flow chart.

A systematic literature review according to Preferred Reporting Items for Systematic Reviews and Meta-Analyses (PRISMA) guidelines was conducted (**Figure 1**) in this study (11). An electronic search on Scopus, Cochrane Library and MEDLINE *via* PubMed database was performed using the following keywords: “minimal invasive surgery”, “minimally invasive surgery”, “MISS”, “MIS”, “conventional open surgery”, “traditional open surgery”, “open surgery”, “spinal metastasis”, “spine metastasis”, “vertebral metastasis”, “spinal metastatic disease” and their MeSH terms in any possible combinations using the logical operators “AND” and “OR”. The reference lists of relevant studies were forward screened to identify other studies of interest. The search was reiterated until October 3, 2021. The review protocol started on September 10, 2021 was registered on the International Prospective Register of Systematic Reviews (PROSPERO), ID: CRD42021283003.

### Inclusion and Exclusion Criteria

In the present review, the full-text English written articles reporting comparisons of PPSF versus OPIF in patients with SM were considered eligible. No date of publication limits were set. Study with follow up shorter than 60 days were excluded from analysis. Expert opinions, studies on animals, unpublished reports, *in vitro* investigations, case reports, case series, letters to the editor, abstracts from scientific meetings and book chapters were excluded from review. The inclusion and exclusion criteria were summarized in **Table 1**.

**Table 1:** Inclusion and exclusion criteria.

Inclusion criteria	Exclusion criteria
English languages	Expert opinions or letters to the editor
Comparative studies between PPSF versus OPIF in patients with spinal metastasis	studies on animals or *in vitro* investigations
Full text article available	unpublished reports or abstracts from scientific meetings
	case reports, case series
	book chapter
	Follow up shorter than 60 days

PPSF, Percutaneous pedicle screw fixation; OPIF,open posterior instrumented fusion.

### Review Question

The review questions were formulated following the PICO scheme (12) (population (P), intervention (I), comparison (C), and outcome (O)) as follows:

Do the patients affected by spinal metastases (P) treated with PPSF surgery (I) have better clinical and functional outcomes (minor blood loss, surgical pain and complication) (O) compared to those treated with OPIF (C)?

### Data Extraction

Two independent authors (A.P. and R.V.) performed the title and abstract screening and collected data from the included studies. Any discordances were solved by consensus with a third author (A.S). The following data were extracted: demographic features, primitive cancer, level involved, Tokuhashi score, Frenkel or American Spinal Injury Association (ASIA) score, intraoperative blood loss, operative time, length of stay, clinical and functional outcomes, possible complications, and follow-up.

### Statistical Analysis

Numbers software (Apple Inc., Cupertino, CA) was used to tabulate the obtained data. Categorical variables are presented as frequency and percentages. Continuous variables are presented as means and standard deviation. Only one decimal digit was reported and was rounded up.

The mean difference (MD) and odds ratio (OR) with 95% confidence interval (CI) were used for each relevant outcome measure. The measured outcomes were presented as a Forest plot. The χ2 test was used to evaluate the heterogeneity between included studies. The I^2^ statistic was performed to estimate the proportion of total variation among analyzed studies; a value higher than 50% was interpreted as substantial heterogeneity. When a large value of I^2^ was obtained a random-effect model was tested, else a fixed-effect model was used. The publication bias was analyzed, according to the MOOSE criteria (**Table 2**) by creating a funnel plot for each outcome analyzed, analyzing its asymmetry. Review Manager Version 5.4.1 (Cochrane Collaboration, Software Update, Oxford, United Kingdom) was used for statistical analysis and generation of Forest plots.

**Table 2:** Results of MOOSE assessment for quality of evidence and risk of bias assessment for the included studies Y, yes; N, no.

	Chi et al., 2021 (13)	Zhu et al., 2021 (14)	Morgen et al., 2020 (15)	Saadeh et al., 2019 (16)	Hikata et al., 2017 (17)	Hansen-Algenstaedt et al., 2017 (18)	Kumar et al., 2017 (19)
Clear definition of study population?	Y	Y	Y	Y	Y	Y	Y
Clear definition of outcomes and outcome assessment?	Y	Y	Y	Y	Y	Y	Y
Independent assessment of outcome parameters?	N	N	N	N	N	N	N
Sufficient duration of follow-up?	Y	Y	Y	Y	Y	Y	Y
No selective loss during follow-up?	Y	Y	Y	Y	Y	Y	Y
Important confounders and prognostic factors identified?	Y	N	Y	Y	Y	N	Y

## Results

### Study Selection

The electronic research of the literature consisted of 1123 studies. Duplicates and non-English articles were removed. Screening by titles and abstracts was performed with subsequent full text reading of the remaining articles. A total of 7 studies met our inclusion criteria and fulfil the purpose of the review (13–19). One of the eligible study was excluded for a short follow up (30 days) (20). The patients included in the meta-analysis were 448, 253 were in the OPIF group whereas 195 were treated with PPSF. **Table 3** summarizes the main characteristics of the included studies such as year of publication, study design and Level of Evidence (LoE), population and recorded variables, type of procedure and instrumented levels. **Table 4** reports primary lesions and the SM locations as well as some demographic data. The mean age of the included patients was 60.7 and the M/F ratio was 1.09 with no differences between the two groups. The most frequent primary lesion was breast, followed by lung and liver.

**Table 3:** Baseline characteristics of the included studies; open posterior instrumented fusion (OPIF) and percutaneous pedicle screw fixation (PPSF).

Author	Year	Study design	Level of evidence (1 – 5)	Period of study	Treatment (Open/MIS)	Number of patient	Male	Female	Age
**Chi EJ** (13)	2021	Retrospective choort study	3	2014-2019	OPIF	29	20	9	61.74 ± 14.72
					PPSF	21	15	6	66.94 ± 10.92
**Zhu X** (14)	2021	Retrospective choort study	3	2017-2019	OPIF	105	65	40	54,1 (26–75)
					PPSF	49	21	28	53.85 (12–82)
**Morgen SS** (15)	2020	Prospective Trial	2	2014-2017	OPIF	26	43%	57%	67.6 (range=42-88)
					PPSF	23	38%	62%	65.9 (range=49-85)
**Saadeh YS** (16)	2019	Retrospective choort study	3	2003-2017	OPIF	20	12	8	60.3 ± 10.9
					PPSF	20	9	11	56.4 ± 9.9
**Hikata T** (17)	2017	Retrospective choort study	3	2009-2015	OPIF	25	12	13	62.8 ± 13.2
					PPSF	25	15	10	63.6 ± 16.0
**Hansen-Algenstaedt N** (18)	2017	Prospective propensity score-matched study	2	2008-2010	OPIF	30	18	12	60.2 ± 15.1
					PPSF	30	13	17	61.8 ± 11.5
**Kumar N** (19)	2017	Prospective cohort study	2	2011-2015	OPIF	18	8	10	65 (49–84)
					PPSF	27	15	12	62 (50–78)

**Table 4:** Patients features and peri-operative data (complications, surgery, blood loss).

Author	Treatment (Open/MIS)	N° of patient	Primary lesion	Level of lesion	Operative time (min)	Blood loss (ml)	Instrumented levels	Decompression	Transfusions (n° of patients)	Length of stay (days)	Complications	Reinterventions
**Chi EJ** (13)	OPIF	29	Liver (6)	T3 (1), T4 (2), T5 (4), T6 (3), T7 (2), T8 (2), T9 (4), T10 (4), T11 (5), T12 (5), L1 (7), L2 (6), L3 (4), L4 (3), L5 (2)	181.47 ± 40.77	696.55 ± 519.43	not specified (“one or two levels”)	yes	–	25.35 ± 20.65	17.2%:	5
			Lung (9)								4 surgical site infection	
			Prostate (2)								1 vertebral body oozing	
			Thyroid (1)									
			Kidney (1)									
			Breast (3)									
			Gastrintestinal (2)									
			Others (5)									
	PPSF	21	Liver (4)	T3 (1), T4 (2), T5 (1), T6 (2), T7 (1), T8 (1), T9 (3), T10 (2), T11 (4), T12 (3), L1 (2), L2 (5), L3 (8), L4 (4), L5 (2)	143.56 ± 49.44	116.67 ± 109.92	not specified (“one ore two levels”)	no decompression	–	11.90 ± 9.69	4%, 1 diplegia after surgery	1
			Lung (7)									
			Prostate (2)									
			Thyroid (2)									
			Kidney (0)									
			Breast (0)									
			Gastrintestinal (5)									
			Others (1)									
**Zhu X** (14)	OPIF	105	Breast (18), Lung (19), Kidney (8), Liver (12), Thyroid (4), Myeloma (4), Colorectal (4), Unknown (9), Prostate (4), Nasopharynx (5), Uterus (2), Other (16)	Thoracic (82), Lumbosacral (23)	221.03	950.48	minimum two levels above and below the lesion	yes	–	9.94	10 Total, 2 Dural tears, 1 brain metastases, 0 Wound hematoma, 6 Wound infection, 1 Early death	–
	PPSF	49	Breast (12), Lung (9), Kidney (7), Liver (2), Thyroid (3), Myeloma (3), Colorectal (1), Unknown (3), Prostate (2), Nasopharynx (3), Uterus (1), Other (3)	Thoracic (36), Lumbosacral (13)	213.45	748.57	minimum two levels above and below the lesion	yes	–	7.35	3 Total, 1 Dural tears, 0 brain metastases, 1 Wound hematoma, 1 Wound infection, 0 Early death	–
**Morgen SS** (15)	OPIF	26	Lung (6), Breast (7), Prostate (1), Unidentified (1), Renal (3), Pancreatic (0), Melanoma (1), Thyroid (1), Lymphoma (0), Other (3)	Thoracic (20), Lumbar (6)	103	500	Two levels above and two below	yes	–	–	2	2
	PPSF	23	Lung (3), Breast (9), Prostate (4), Unidentified (4), Renal (2), Pancreatic (1), Lymphoma (3),	Thoracic (17, Lumbar (6)	142	175	Two levels above and two below	yes	–	–	2	2
**Saadeh YS (** 16 **)**	OPIF	20	Breast (20), Colon (10), Lung (20), Melanoma (4) Renal (10), Squamous cell (10), Other (25)	Cervicothoracic (10), Thoracic (40), Thoracolumbar (20), Lumbar (30);	266	1732 ± 359	5.7 ± 1.8	yes	50	8.3 ± 1.4	8 total (2 DVT, 1 PE, 2 Thrombocytopenia, 1 Wound complication, 1 Durotomy, 1 Mortality (30 day))	2
	PPSF	20	Breast (20), Colon (10), Lung (20), Melanoma (5), Renal (10), Squamous cell (10), Other (25)	Thoracic (40), Thoracolumbar (55), Lumbar (5)	296	805 ± 138	6.2 ± 2.2	yes	30	8.3 ± 1.7	8 total (1 DVT, 0 PE, 2 Respiratory complication, 1 Wound complication, 1 Heart failure, 1 Acute kidney injury, 1 Durotomy,	3
**Hikata T** (17)	OPIF	25	Lung (2), Thyroid (6), Breast (4), Kidney (3), Liver (3), Lymphoma (1), Rectus (1), Uterus (1), Larynx (1), Myeloma (1), Unknown (2)	Thoracic (18), Lumbosacral (7)	188.9 ± 43.6	714.3 ± 545.9	8.1 +- 2.9	yes	–	–	7 Massive bleeding (>1000 mL), 2 Wound hematoma, 2 Neurological deficit	–
	PPSF	25	Lung (7), Thyroid (2), Breast (3), Kidney (2), Liver (1), Prostate (4), Lymphoma (2), Rectus (1), Gastric (1), Rhabdomyosarcoma (1), Melanoma (1)	Thoracic (17), Lumbosacral (8)	204.6 ± 55.4	340.1 ± 302.5	8.3 ± 2.4	yes	–	–	1 Massive bleeding (>1000 mL), 1 Wound hematoma, 1 Neurological deficit,	–
**Hansen-Algenstaedt N** (18)	OPIF	30	Breast (4), Prostate (8), Lung (3), Thyroid (4), Renal (1), Gastrointestinal (1), Others (5)	–	220.4 ± 57.9	2062.1 ± 1148.0	3.8 ± 1.7	yes	23	21.1 ± 10.8	3 Wound infection, 2 Dural tear, 3 Neurological, 2 Lung infection, 2 Urinary tract infection	–
	PPSF	30	Breast (14), Prostate (3), Lung (5), Thyroid (1), Gastrointestinal (4), Others (3)	–	190.9 ± 78.4	1156 ± 572.3	5.5 ± 3.1	yes	12	11.0 ± 5.0	2 Dural tear, 2 Neurological, 1 Lung infection, 2 Urinary tract infection	–
**Kumar N** (19)	OPIF	18	Lung (5), Breast (5), Gastrointestinal (1), Prostate (5), HCC (1), Other (1)	–	269	961	–	yes	–	13	16%	–
	PPSF	27	Lung (7), Breast (3), Gastrointestinal (2), Renal (2), Prostate (1), Lymphoma (3), HCC (2), Others (3)	–	253	184	–	yes	–	9	3%	–

Open posterior instrumented fusion (OPIF) and Percutaneous pedicle screw fixation (PPSF), Deep Vein Thrombosis (DVT), Pulmonary Embolism (PE).

The number of instrumented levels was not always specified. In five of the included studies, patients had posterior pedicle screw instrumentation of two levels above and two levels below the metastatic lesion at least. Data about that were inhomogeneous as two articles even reported the number of instrumented levels.

Among the included articles, the mean reported follow-up period was 16.2 months. The longest one was that by Kumar N et al. which last up to five years (19).

### Complications

**Figure 2:**
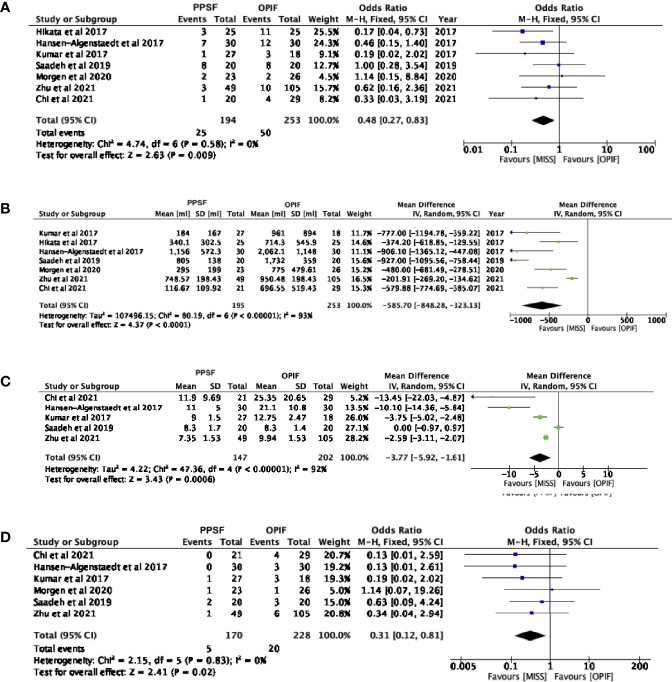
Forest plots comparing surgical outcomes between Open posterior instrumented fusion (OPIF) and Percutaneous pedicle screw fixation (PPSF). **(A)** Complications, **(B)** blood loss **(C)** hospital stay, **(D)** infection rate. SD, standard deviation; IV, inverse variance; CI, confidence interval.

All the included studies reported postoperative complications, with 195 patients in the PPSF group and 249 in the OPIF group (13–19). The meta-analysis of these data showed an odds ratio of 0.48 (95% CI, 0.27 to 0.83; p= 0.009), showing a decreasing odd of complications in the PPSF group compared to OPIF (**Figure 2A**).

### Blood Loss

All the included studies reported intraoperative blood loss, with 195 patients in the PPSF group and 253 in the OPIF group (13–19). The meta-analysis of the data revealed a mean difference of -585.70 (95% IC, -848.28 to -323.13; p< 0.0001), thus suggesting a decreasing odd of complications in the PPSF group compared to OPIF (**Figure 2B**). Transfusions were reported in two studies only (16, 18)

### Hospitalization

The length of hospitalization was reported in 5 of the included papers, with 147 patients in the PPSF group and 202 in the OPIF group (13, 14, 16, 18, 19). The meta-analysis of the data revealed a mean difference of -3.77 (95% IC, -5.92 to -1.61; p= 0.0006), thus suggesting a decreasing mean hospital stay in the PPSF group compared to OPIF group (**Figure 2C**).

### Infections

Six of the included studies reported the occurrence of postoperative infections, with 170 patients in the PPSF group and 228 in the OPIF group (13–16, 18, 19). The meta-analysis of these data showed an odds ratio of 0.31 (95% CI, 0.12 to 0.81; p= 0.02), showing a reduced infection rate in the PPSF group compared to the OPIF group (**Figure 2D**).

### Reinterventions

**Figure 3:**
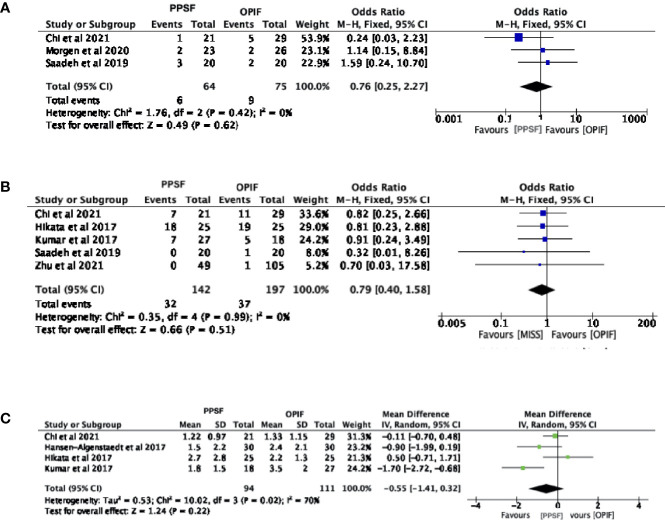
Forest plots comparing clinical outcomes between Open posterior instrumented fusion (OPIF) and Percutaneous pedicle screw fixation (PPSF). **(A)** Reintervention, **(B)** mortality, **(C)** postoperative pain. VAS, Visual Analogue Scale; SD, standard deviation; IV, inverse variance; CI, confidence interval.

A total of three studies described the occurrence of reinterventions, with 64 patients in the PPSF group and 75 in the OPIF group (13, 15, 16). The meta-analysis of these data showed an odds ratio of 0.76 (95% CI, 0.25 to 2.27; p= 0.62), suggesting that both techniques demand a similar reintervention rate (**Figure 3A**).

### Mortality

A total of six studies described mortality, with 142 patients in the PPSF group and 197 in the OPIF group (13, 14, 16, 17, 19). The meta-analysis of these data showed an odds ratio of 0.79 (95% CI, 0.40 to 1.58; p= 0.51), demonstrating that both techniques have a similar mortality rate (**Figure 3B**).

### Pain

Among the included studies, four articles described pre and post-operative VAS, with 103 patients in the PPSF group and 102 in the OPIF group (13, 17–19). The meta-analysis of the preoperative VAS data revealed a mean difference of -0.03 (95% CI, -0.30 to 0.25; p= 0.84), whereas the mean postoperative VAS difference was -0.55 (95% CI, -1.41 to 0.32; p= 0.22). The meta-analysis suggested that both techniques have a similar postoperative VAS (**Figure 3C**).

### Clinical Outcomes and Survival

An heterogeneous set of scores was applied to assess preoperative health status and clinical outcomes but none of them was used in each of the included studies. Therefore, a statistical analysis was not possible. Preoperative evaluation of metastatic spine tumor prognosis was measured by using the Tokuhashi scoring system in half of the selected papers (15, 17–19). The Frankel Scale for spinal cord injuries was employed in four of the included studies to classify the pre- and postoperative extent of the neurological and functional deficit (14, 17–19). Many other scores were employed in search strategies, such as Oswestry Disability Index (ODI), American Spinal Injury Association (ASIA) Impairment Scale and Spinal instability neoplastic score (SINS), Tomita score, the Karnofsky performance scale index, etc. Also survival was not always specified. In fact, a total of four studies described the mean survival time but as data were presented in heterogeneous forms, it was not possible to perform a statistical analysis.

### Surgical Decompression

Both techniques, PPSF and OPIF, allow for a decompression of neurological structures. In fact conventional open or mini-open decompression was performed in all the included cases except for the PPSF group by Chi E J et al. (13). The OPIF procedure, with a midline incision and a large dissection of paraspinal muscle, allows wider neurological decompression, and major possibility of tumour lesion debulking (14, 15). On the other hand to obtain a satisfying decompression with PPSF approach, various techniques were described. In the case of a unilateral tumour spinal cord compression, the same paramedian access for screw placement could be used, through the use of dedicated retractors, for decompressive manoeuvres (18).

While in case of a 180 degree compression a midline mini-open could be performed with the possibility to obtain a sufficient posterior decompression (14–18). An hybrid approach could be the choice in cases that requires long fixation and wide neurological decompression (9).

### Response to Review Question

SM patients treated with PPSF compared to those treated with OPIF have a lower rate of complications and infections, less intraoperative blood loss and a shorter hospital stay. A doubt still remains about mechanical complications, short and medium-term survival and post-operative pain.

### Bias Assessment

A risk of bias assessment was performed by the MOOSE criteria of included study as reported in **Table 2**. No obvious bias risk was found for the included study. A funnel plot for all analyzed outcomes was obtained. Nevertheless, no significant asymmetry was found.

## Discussion

There is a growing interest in managing SMs because of their crucial clinical implications in oncological patients and their consequent increasing social and economic burden (21). The spine represent the most common localization of bone metastasis, accounting for about 50% of all the secondary malignant growths (22, 23). Moreover, up to 20% of these patients will experience metastatic spinal cord compression (MSCC). This is an oncological emergency characterized by severe spinal pain increased by load and impaired neurological function (limb weakness, difficulty walking, sensory loss, bladder or bowel dysfunctions) (24).

In recent years, cancer survival improved for all of the most common malignant tumours just as the mortality rate has decreased, indicating a progression in fight against cancer due to prevention, early detection and new treatment innovations (25).

The prognosis and the mean survival in SMs patients essentially depends on the primary tumour biology. A longest survival was reported in patients with haematological malignancies and prostate cancer compared to those with lung cancer (26, 27). Notwithstanding only 10% to 20% of patients with SMs are still alive two years after the diagnosis of metastatic disease (5).

Besides, the QoL of these patients is often not impaired by cancer. Hence, when a surgical treatment is indicated, an interdisciplinary evaluation of the patient’s overall disease situation should be performed and the target of the treatment planning should be the preservation of the QoL, shifting the treatment goals from cure to palliation (28). The aim of surgery is: (I) neurological impairment prevention by posterior or anterior decompression (laminectomy and hemi-facetectomy), (II) reduction of tumour volume or tumour debulking and (III) stabilize the affected spinal segment to allow the patient mobilization safely without bracing (5).

Up to 25% of patients who undergo conventional open surgery for SMs present perioperative complications (29, 30). During the last decade, PPSF appeared to be an appealing alternative for the management of spinal fractures (31–33) and its advantages became early attractive for the stabilization in spinal metastatic disease (9). Minimally invasive approaches for posterior spinal fixation allows minor intraoperative blood loss, an earlier adjuvant therapy, and a shorter overall hospital stay (32). On the other hand, by using OPIF techniques, posterior elements from the vertebra above to the vertebra below the involved segments are exposed, resulting in extensive damage to back muscles and soft tissues with delay of mobilization and prolonged hospital stay (32)

An earlier mobilization avoids the complications linked to bed rest such as muscular mass loss and sarcopenia, constipation, altered ventilation/perfusion, deep vein thrombosis and pulmonary embolism (32). Furthermore, a shorter hospitalization reduces the exposure of the patients to infectious disease, especially during the present SARS-cov2 pandemic (34). A faster postoperative recovery and a poor need for care reduces economic burden and present and a significant psychological and social impact.

Moreover, PPSF permit to avoid the back muscle detachment and retraction, which causes postoperative pain and profuse bleeding, thus reducing intraoperative blood loss and consequent demand for transfusions (28). Furthermore, smaller incisions reduce the wound complications and offer a better aesthetic outcomes (35).

The abovementioned advantages are crucial in preserving and improving the QoL of oncological patients with poor midterm life expectancy.

In patients with metastatic spinal disease, meta-analysis of the available data revealed that PPSF is associated with a statistically significant reduction of blood loss, postoperative complications, infection, and hospitalization when compared with OPIF. Above all, the reduction of infections plays an important role in the management of these patients who present themselves at an increased risk of infection due to tumour-induced immune suppression and radio-chemotherapy which could reduce the wound healing ability.

Furthermore, even if it was not statistically significant, PPSF revealed lower post-op VAS rates compared to OPIF group, suggesting that percutaneous procedures may have better results in terms of pain. Moreover, PPSF was not inferior to OPIF with respect to mortality and reintervention.

Our results suggest comparable rates of perioperative surgery-related complications between the study groups, confirming the safety of the PPSF technique.

No implant failures or other mechanical complications such as septic or aseptic loosening were reported in both study groups. However, the mean follow-up of the included studies was short, which does not permit further consideration on implant loosening.

Few studies considered the QoL of surgically treated oncological patients with SMs. Therefore, we believe that clinical outcomes measurement will be a major topic of interest for future studies in order to determine which of the above-mentioned surgical treatments achieve the best QoL preservation and improvement with the lowest number of complications.

### Clinical Implication

Patients treated with PPSF, due to fast clinical recovery and surgical wound healing, could resume or start faster chemo- and radiotherapy than those treated with OPIF. This could play a crucial role in determining patient survival and local disease control (17).

The results of this meta-analysis suggest a minimal superiority of the PPSF treatment compared to OPIF in SMs patients who require spinal stabilization with or without neurological decompression. Therefore, PPSF should be considered the first-line choice in these patients if there are no contraindications.

Relative contraindications could be: (I) more than 6 levels of spinal fusion, (II) need for extensive neurological decompression, (III) correction of important post traumatic deformities.

### Limitation

This meta-analysis is not without limitations. First of all, all included studies are observational studies except one which is a randomized clinical trial. Secondly, the number of included studies is not large enough to perform a meta-regression analysis. Finally, the data of some studies on certain outcomes are too inhomogeneous to be able to perform an accurate analysis of the data.

## Conclusion

The PPSF treatment is associated with fewer intra and peri-operative complications, a lower rate of infections, a reduction in intraoperative blood loss and a shorter hospital stay compared to the OPIF treatment. PPSF treatment should be used whenever possible for palliative surgery in SM patients. Studies focused on the patient’s quality of life and randomized clinical trials are however necessary to provide superior quality scientific evidence.

## Data Availability Statement

The raw data supporting the conclusions of this article will be made available by the authors, without undue reservation.

## Author Contributions

AP, AS, FT and GM contributed to conception and design of the study. AS and CV organized the database. performed the statistical analysis. AP, AS, RV wrote the first draft of the manuscript. CV, LP and FT wrote sections of the manuscript. All authors contributed to manuscript revision, read, and approved the submitted version.

## Funding

Publication costs are founded by the Catholic University of Sacred Heart, department of Orthopedics and Traumatology.

## Conflict of Interest

The authors declare that the research was conducted in the absence of any commercial or financial relationships that could be construed as a potential conflict of interest.

## Publisher’s Note

All claims expressed in this article are solely those of the authors and do not necessarily represent those of their affiliated organizations, or those of the publisher, the editors and the reviewers. Any product that may be evaluated in this article, or claim that may be made by its manufacturer, is not guaranteed or endorsed by the publisher.
